# Continuous Compression Implants in Foot and Ankle Surgery: Tips and Tricks

**DOI:** 10.3390/jcm14103507

**Published:** 2025-05-16

**Authors:** Konstantinos Tsikopoulos, Konstantinos Sidiropoulos, Dimitrios Kitridis, Constantinos Loizou, Alisdair Felstead

**Affiliations:** 1 Orthopaedic Department, North Bristol NHS Trust, Bristol BS10 5NB, UK; 2 Orthopaedic Department, Florina General Hospital, 531 00 Florina, Greece; kcdroq@yahoo.gr; 3 Orthopaedic Department, 424 General Military Hospital, 564 29 Thessaloniki, Greece; dkitridis@gmail.com; 4 Orthopaedic Department, Oxford University Hospitals NHS Trust, Oxford OX3 9DU, UK; constantinos.loizou@ouh.nhs.uk; 5 Orthopaedic Department, Portsmouth Hospitals NHS Trust, Portsmouth PO6 3FT, UK; alisdair.felstead@porthosp.nhs.uk

**Keywords:** continuous compression implants, staples, midfoot/hindfoot fusion, Lisfranc, surgical technique

## Abstract

**Background:** Continuous Compression Implants (CCIs) are low-profile implants made of nitinol and titanium. They offer multiple benefits in comparison to plate and screw fixation for foot and ankle indications, and they are designed in such a way that they continuously and dynamically compress the opposed bony surfaces throughout the entire healing process. **Methods:** In this study, we present our experience on the use of those nitinol implants for midfoot and hindfoot surgery. Furthermore, we elaborate on the advantages and downsides of using this internal fixation method and highlight common pitfalls which could lead to undesirable clinical outcomes. We also demonstrate our proposed surgical technique on how to use CCIs in a reproducible and reliable way and present surgical tips which could help reduce surgical time when utilising these implants. We also make surgical recommendations on their use and present the underlying biomechanics, which could provide a better understanding of the rationale behind using them in the field of foot and ankle surgery. Last but not least, we presented the early clinical and radiological results of a series of patients who underwent primary midfoot fusion for Lisfranc injury between 2020 and 2023. **Results:** With a minimum follow-up of 9 months, satisfactory clinical and radiological union was noted in all those patients. The mean difference between pre- and post-operative MOxFQ scores was −37.7 (95% CI was 16.9 to 58.5; *p* = 0.03). The mean post-operative VAS pain at rest was 3.2 (SD = 2.3). No major complications were noted. **Conclusions:** CCI internal fixation is a safe, reproducible, and reliable method when it comes to foot and ankle conditions, but it requires appropriate pre-operative planning, surgical training, and careful implantation.

## 1. Introduction

### Definition

Nitinol Continuous Compression Implants (CCIs) are low-profile implants manufactured from nitinol and titanium (approximately at a 50/50% ratio). They feature pseudo-elastic properties and shape-memory in addition to good corrosion resistance, no cytotoxic, genotoxic, or allergic activity, and excellent biocompatibility [1]. The word “ni-ti-nol” is derived from the metals utilised, that is, nickel and titanium, in addition to “nol”, which stands for Naval Ordnance Laboratory, as the U.S. military first utilised this biomaterial in 1965 along with W.J. Buehler. CCIs are designed in such a way that they continuously and dynamically compress the opposed bony surfaces throughout the entire healing process [1]. The ultimate goal of utilising these implants is to minimise the effects of bone resorption and maintain a stable construct. Also, another rationale is to recover compression after repetitive loading.

From an evolutionary standpoint, static staples were first utilised, and they were made of stainless steel or titanium. They only provided minimal compression (if any), and they were prone to distal gapping. Subsequently, the first nitinol staples were body temperature-activated, and they required freezer storage and heating prior to activation. On the contrary, the new-generation super-elastic nitinol compression staples are ready to use at room temperature, and no activation is needed.

It is worthy of note that CCIs could potentially be advantageous over traditional fixation methods since they enable smaller surgical incisions, decrease surgical time compared to plate and screw fixation, provide continuous compression that leads to satisfactory union rates post-operatively, and cause less irritation in the midfoot/hindfoot area where there is limited overlying soft tissue due to their low profile [2]. Nevertheless, we wish to highlight that the continuous compression forces provided by the CCIs cannot compensate for inappropriate joint preparation when it comes to midfoot/hindfoot fusions.

When compared to other established methods of fusion fixation, such as the crossed-screw and plate-and-screw techniques, comparable biomechanical performance has been documented [1,3]. In more detail, nitinol staples demonstrate increased compression surface area and the improved recovery of plantar gaping after mechanical loading when compared to plate-and-screw constructs [4,5]. Moreover, an activated Nitinol implant self-adjusts over time to continuously and dynamically compress together the opposing bony surfaces. To elaborate further, the implant initially comes in a closed position, which provides secure fixation and resists migration, with the bowing bridge allowing for the even distribution of the compression forces along the near and far cortices. Consequently, when the implant is loaded in an open position, the compression forces are stored similar to a powerful spring. During the implantation phase, the implant is released in bone, which in turn allows for dynamic compression as the staple attempts to return to its initial closed design shape. It should be noted that CCIs come at a relatively high cost compared to plate and screw fixation, and therefore, the accurate placement of the implants is recommended as they can only be deployed once.

## 2. Materials and Methods

Institutional review board approval was obtained, and the data utilised in this paper were retrospectively collected to review the efficacy and complications following fusion surgery for foot and ankle indications. The Elite BME (Synthes USA, LLC, Monument, CO, USA; or Bio-Medical Enterprises, Inc., San Antonio, TX, USA) and SpeedTitan continuous compression staples (Synthes USA, LLC, Monument, CO, USA; or Bio-Medical Enterprises, Inc., San Antonio, TX, USA) were used in a single institution by two fellowship-trained foot and ankle surgeons between 2021 and 2023. Inclusion criteria were patients between the ages of 18 and 85 years of age requiring fusion for foot and ankle conditions such as midfoot/hindfoot arthrosis and acquired flat foot deformity. We excluded diabetic patients and advanced osteoporosis. A subset of patients requiring Lisfranc fusion was further analysed from a clinical and radiological point of view. The primary outcome was the clinical and radiological union. The secondary outcomes included the assessment of functional disability as measured with the Manchester–Oxford Foot Questionnaire (MOxFQ), pain at rest as expressed with a visual analogue scale (VAS), and post-operative complications. The included patients were randomly selected from the local hospital database using computerised software. For the case series on Lisfranc fusions presented in this paper, 41 patients were assessed for eligibility, and 6 of those were considered in the analysis. A minimum of 9 months follow-up was considered.

For the midfoot fusions presented in this paper, early clinical and radiological results were evaluated. The primary outcome was the clinical and radiological union. The secondary outcomes included the assessment of functional disability as measured with the Manchester–Oxford Foot Questionnaire (MOxFQ), pain at rest as expressed with a visual analogue scale (VAS) at rest, and post-operative complications. The statistical analysis was performed with the SPSS software (Version 27.0. Armonk, NY, USA: IBM Corp.) and a *p* value of 0.05 indicated statistical significance.

### 2.1. Surgical Technique

For the joint preparation, a combination of chisels, osteotomes, and curettes was utilised. This was followed by the appropriate fenestration of the subchondral plate with drill bits, and bone grafting was utilised as required at that stage.

**Figure 1:**
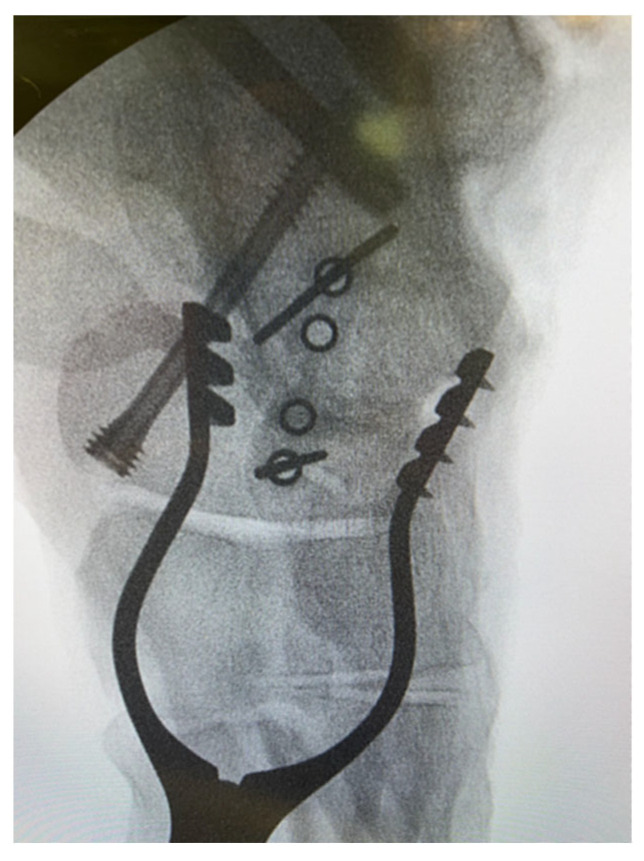
Intra-operative image of talonavicular joint fusion demonstrating the ‘perfect circle technique’.

We highlight that the ‘perfect circle technique’ was implemented prior to introducing the implants to avoid undesirable joint penetration, which would inevitably compromise the compression across the fusion site and result in pain and patient dissatisfaction. This ‘perfect circle technique’ involved the use of the staple guide along with the corresponding staple guidewires. This construct was then screened with the image intensifier, and the radiograph was considered satisfactory when the holes on either side of the staple guide were perfectly superimposed (Figure 1).

This translates to an image of a round circle in the guide rather than an ellipse. To quickly achieve perfect circles without specific instrumentation, the surgeon should manipulate the position of the foot in relation to the drill guide, which remains in place throughout the wire and subsequent pin placement. Moreover, a true lateral view is highly recommended for the surgeon to confirm that the position of the wires is not intra-articular. To effectively prevent this complication from happening, the surgeon should be mindful of the orientation of the joints (e.g., saddle-shaped calcaneocuboid joint).

### 2.2. Results Following Primary Fusion for Lisfranc Injuries

We retrospectively identified 6 adult patients (mean age and BMI were 46 years and 33.4 kg/m^2^, respectively) who underwent primary fusion for their midfoot injuries between 2020 and 2023. With a minimum follow-up of 9 months, satisfactory clinical and radiological union was noted in all patients. The mean difference between pre- and post-operative MOxFQ scores was −37.7 (95% CI was 16.9 to 58.5; *p* = 0.03). The mean post-operative VAS pain at rest was 3.2 (SD = 2.3). No major complications were noted.

## 3. Discussion

### 3.1. Recommendations on the Arm Length, Number of Staples per Joint, and Number of Legs Are Given Below

**Figure 2:**
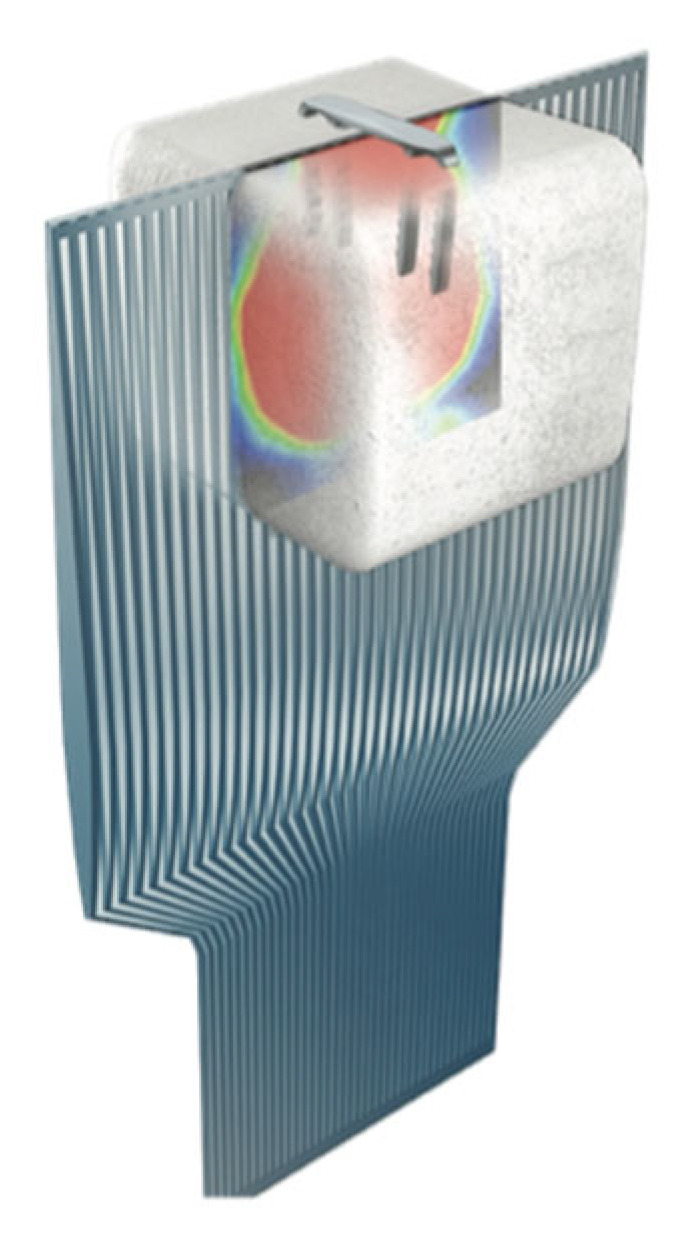
Illustration depicting the compression area, which expands beyond the tip of the continuous compression staple based on biomechanical measurements.

Regarding the ideal length of the arms, evidence has shown that the CCIs enable compression distal to their tip (Figure 2).

Therefore, we advocate that it is safe to use shorter arms than the depth of the bone, particularly when there is a high risk of breaching the adjacent joint, thus causing irritation. In more detail, in terms of the ideal length of the legs, biomechanical evidence has shown that the bicortical placement of nitinol staples is not required to achieve adequate compression. To elaborate further, the same amount of compression can be achieved when placing staples 2 mm short of the far cortex when compared with bicortical placement. Moreover, from a biomechanical point of view, troughing does not appear to affect the properties of the construct. Therefore, the intentional troughing of the bone during implant placement will result not only in less implant prominence but also in no changes to the biomechanical properties/compression capacity of the nitinol staples [6]. In addition, we wish to highlight the fact that although no clear guidelines on the ideal length of the staples exist, evidence has shown that a 20 mm bridge length exhibits higher contact forces and lower stresses as compared to 15 mm bridge length, thus suggesting that the longer the bridge length, the more stable the internal fixation [7].

At present, there are no published guidelines as to how many staples should be used per joint, despite the fact that biomechanical studies have shown that two staples provide a biomechanically sound construct featuring increased stiffness, joint contact area, and peak load [4]. In our experience, compression can be assessed intra-operatively following the introduction of the CCIs, and depending on the subsequent visual and radiographic assessment performed by the surgeon, a further implant could be considered.

**Figure 3:**
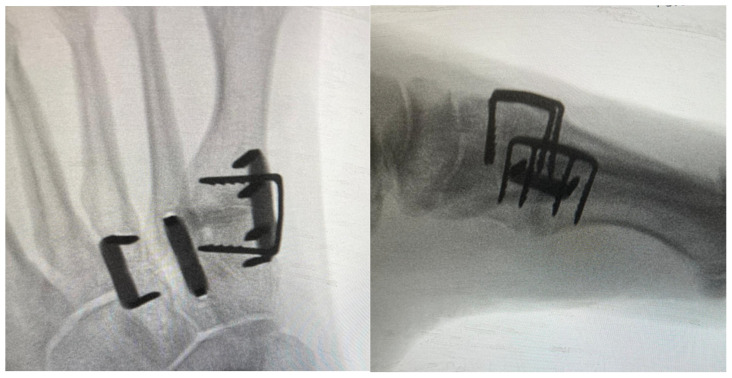
Intra-operative radiographs depicting fusion of tarsometatarsal joints with a combination of two- and four-leg staples.

In terms of the legs of the staples, there are no clear recommendations in the literature at the moment. From a biomechanical perspective, where possible, four-leg staples should be utilised to allow for the even distribution of compression forces through the tips. For instance, this could be the case for the first tarsometatarsal joint [2] (Figure 3). Of note, dual-staple constructs applied in an orthogonal fashion provide greater than doubled compression in comparison to constructs with a single staple. Moreover, double-staple constructs appear to be 2 times stiffer than plate constructs. This finding may have potential implications in clinical practice as double-staple constructs should be used where feasible [5].

### 3.2. Assessment of Union Post-Operatively

For a fusion to be considered radiologically successful, a minimum of 50% healing is required [8]. On top of that, broken hardware is suggestive of a nonunion. From a clinical standpoint, continued pain/discomfort in the subsequent clinic follow-ups denotes clinical nonunion. If in doubt 9 months post-operatively, a CT scan is warranted.

From a radiological standpoint, the majority of studies utilise plain radiography as the main imaging modality when it comes to evaluating the union rates following fusion with continuous compression staples. It is worthy of note that although CT features higher sensitivity and specificity for union detection, only a limited number of authors have implemented it in their papers [9,10].

At this point, we wish to draw the readers’ attention to the fact that a “disguise” nonunion may be seen in patients rarely. This phenomenon occurs when radiographs do not depict the nonunion, given the continuous compression exerted by the continuous compression staples [11]. In other words, one of the downsides of using CCIs could be that nonunion may appear radiographically occult on plain radiographs.

### 3.3. Broken Metalwork and Requirement for Hardware Removal

**Figure 4:**
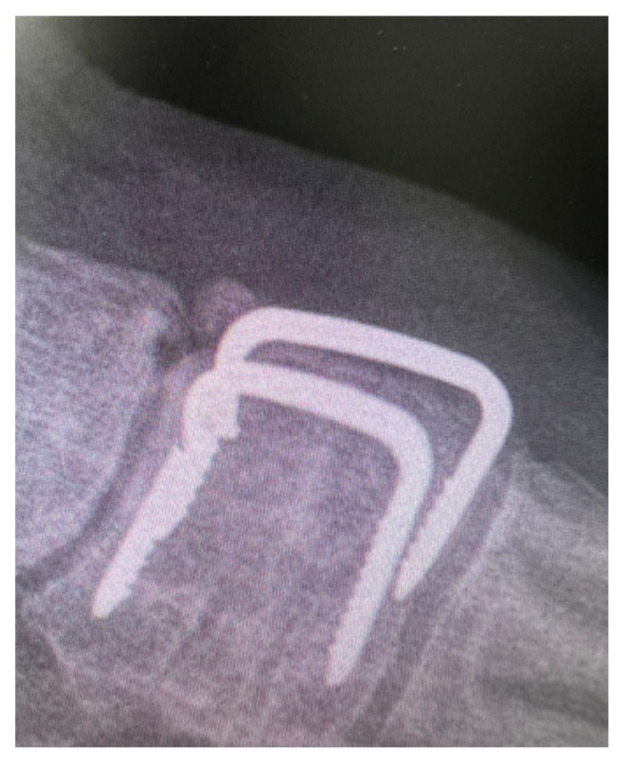
Lateral midfoot radiograph depicting broken metalwork 6 months following tarsometatarsal joint fusion.

Of note, staples with a narrow bridge carry a higher risk of fatigue failure as their bending stiffness appears to be insufficient when it comes to midfoot and hindfoot fusions (Figure 4).

The incidence of nonunion in the presence of broken nitinol staples appears to be high [12]. In terms of the removal of hardware due to secondary pain, it appears that the plate-and-screw constructs present a higher rate in comparison to the staple-only construct [13].

### 3.4. Factors Affecting Biomechanical Performance/Risk Factors for Nonunion

It is undeniable that results following internal fixation with nitinol staples are influenced by the bone quality [14]. Moreover, further risk factors for union include a BMI equal to or greater than 35, the fusion of the Chopart joints (especially in isolation), surgery for diabetic patients, and male patients. Therefore, utilising nitinol staples in isolation in osteoporotic bones is not recommended, and caution should be exercised when considering the application of bone grafting.

### 3.5. Examples of CCI Application for Foot and Ankle Indications

CCIs can be effectively used either alone or in conjunction with other fixation methods (e.g., plates and screws). Moreover, they can be used not only for fusions but also for securing extra-articular osteotomies (e.g., distal metatarsal, Akin, Evans, Cotton).

**Figure 5:**
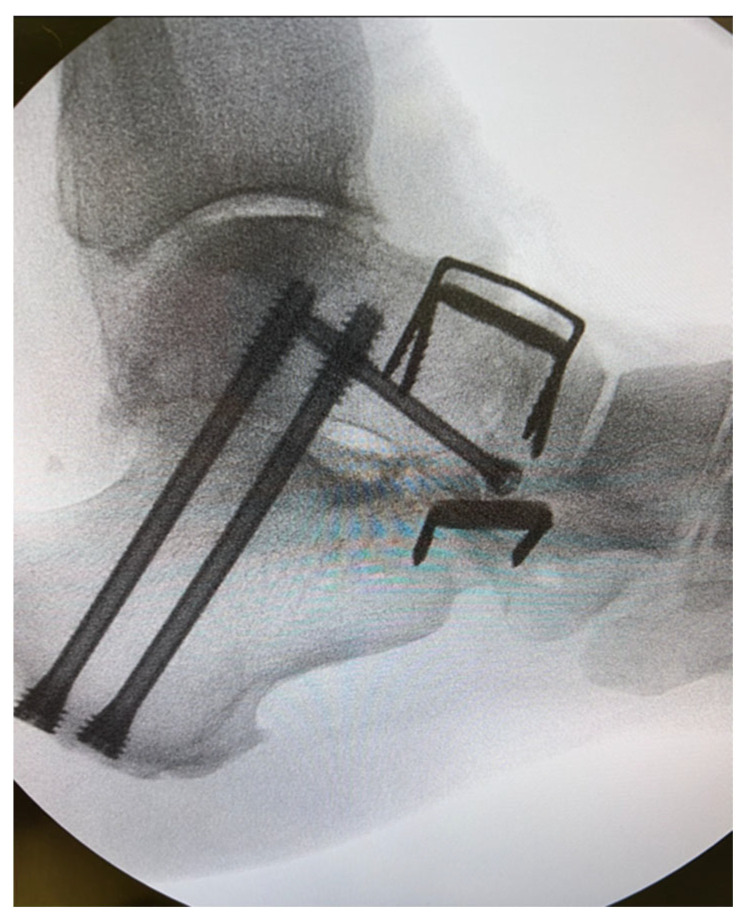
Lateral intra-operative radiograph depicting a realignment triple fusion performed with a combination of internal fixation techniques including CCIs. Note that the dorsal osteophytes at the talonavicular joint were debrided to allow for appropriate staple position, thus preventing anterior ankle impingement.

In our experience, we have found CCIs particularly useful in the setting of a triple fusion as the tourniquet time is of the essence (Figure 5). In more detail, we claim that CCIs could result in less peroneal tendon irritation when compared to plates for a calcaneocuboid fusion. Regarding the talonavicular joint, recent evidence has demonstrated that CCIs exhibit equivalent functional/biomechanical properties when compared to the “gold standard” lag screws [15]. In a recent systematic review with a total of nine articles looking at talonavicular arthrodesis, fusion rates were found to be higher in the staple fixation group (i.e., 100%, n = 13) than in the screw fixation one (n = 75)—87.5% to 100% [16].

**Figure 6:**
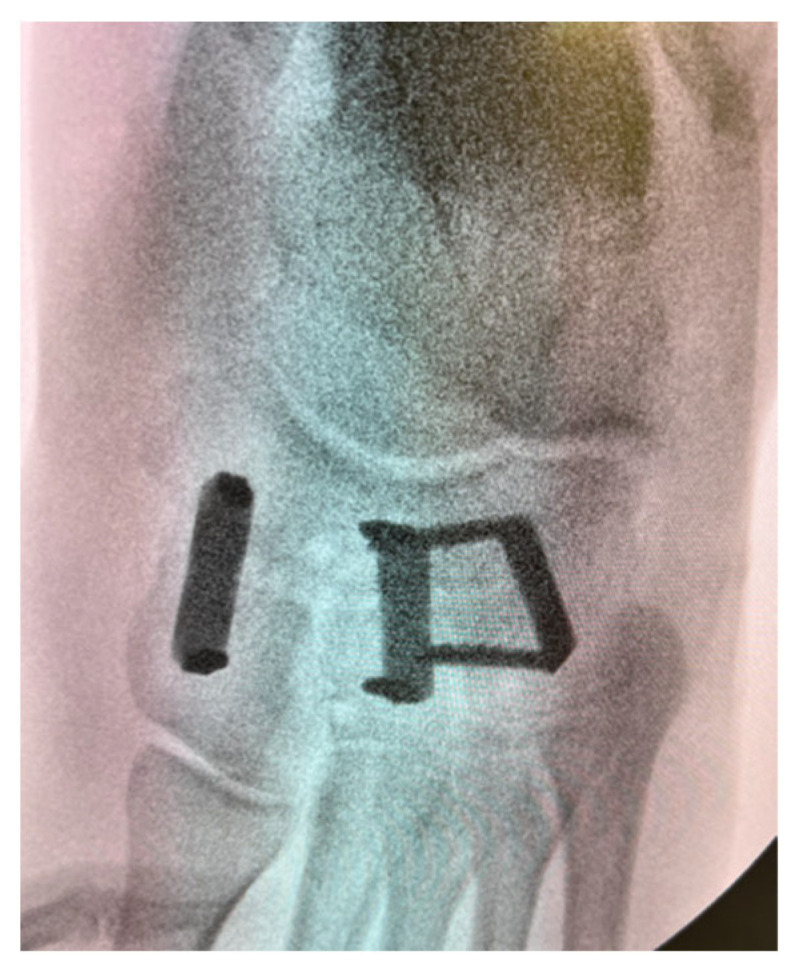
Intra-operative anteroposterior radiograph depicting a naviculocuneiform joint fusion with CCI fixation.

Likewise, based on our experience, we highly recommend the use of CCIs for midfoot fusions, such as naviculocuneiform and tarsometatarsal joints, as irritation from metalwork is less likely compared to plate and screw fixation (Figure 6).

**Figure 7:**
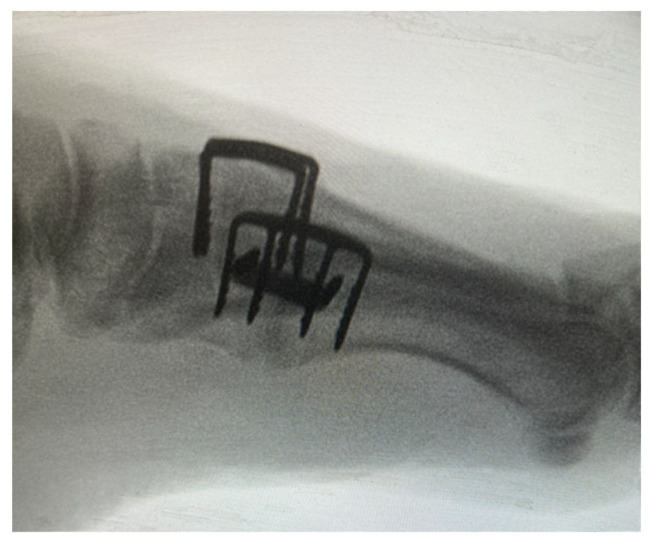
Lateral intra-operative radiograph depicting a 1st/2nd/3rd tarsometatarsal joint fusion with a 90-90-degree configuration technique for the 1st TMTJ.

Regarding the first tarsometatarsal joint, multiple studies have demonstrated that the efficacy of CCIs is satisfactory, with a fusion rate of approximately 90% and a patient satisfaction rate similar to that seen with other fixation methods [17]. In our experience, the 90-90-degree configuration technique yields satisfactory outcomes and essentially requires placement of two staples, one in the dorsal and the second in the medial position (Figure 7). Of note, those two staples should be sequentially tamped down to the bone to ensure that the arms would not interfere with each other, thus compromising compression.

Furthermore, recent evidence on Lisfranc injuries has demonstrated that the use of CCIs for primary arthrodesis could result in shorter tourniquet times and improved union rates when compared to plate-and-screw constructs [13]. In our series of six patients who underwent fusion for their Lisfranc injuries, we demonstrated promising results with minimal complications. Those findings are in line with the most recent literature [13].

Regarding flat foot deformity correction, based on our experience, we claim that implementing CCI fixation for medialising os calcis osteotomy offers multiple advantages compared to screw fixation. In more detail, for the osteotomy to be meaningful, the appropriate amount of translation has to be maintained, and this can be reliably achieved with CCIs, given the unique design of the calcaneal shift staples.

In terms of talonavicular arthrodesis, a cadaveric study has recently demonstrated that fixation with a combination of 5.5 mm cannulated screw with a nitinol compression staple is advantageous over fixation with a single 5.5 mm screw from a biomechanical point of view [18]. In more detail, higher failure load, stiffness, and cycles to failure were noted in the staple–screw group [19].

### 3.6. Relative Contraindications for Nitinol Staples

Concerning the first metatarsophalangeal joint fusion, in our practice, we tend to avoid CCIs given the fact that the biomechanical and clinical evidence on nitinol staple constructs has not been promising as of yet [11,20].

Furthermore, we advise caution when attempting to span multiple joints with nitinol staples and when it comes to comminuted fractures. Finally, we strongly recommend against orthopaedic surgeons using CCIs for osteoporotic patients, as the compression is questionable in these circumstances, thus increasing the risk of nonunion. Based on six published papers, the rate of complications overall was found to be 11.25% [21]. On the other side, when it comes to poor soft tissue quality, staples are an effective alternative. Likewise, when extensive scarring from previous procedures exists, staples might be a viable option.

### 3.7. Removing CCIs

We wish to draw the readers’ attention to the fact that CCIs should not be left proud. Osteophytes and bony prominences are the main culprits in prominent staples, which could then lead to irritation, thus requiring removal. In these circumstances, we highly recommend the careful preparation of the bony bed with rongeurs and/or chisels to achieve an even surface, which will allow for the staples to sit flush with the bone.

From a technical point of view, when planning the removal of CCIs, one should bear in mind that releasing the continuous compression forces prior to metalwork removal results in an uneventful and straightforward surgical procedure. This is because the CCIs continuously attempt to return to their original closed-shape design, thus rendering removal problematic from a biomechanical perspective. Therefore, to achieve an uneventful removal and decrease surgical time, we suggest that surgeons use the large wire-cutting pliers to cut the bridge of the staple after the staple has been slightly lifted off the bone with the use of a small osteotome, thus allowing for the tension to be effectively released. Subsequently, the two remaining staple pieces can be easily extracted with the use of pliers or rongeurs.

### 3.8. Limitations of the Work

This study has some limitations. First of all, it is based on the experience of two orthopaedic centres only. Second, we have not presented any clinical data to demonstrate the clinical efficacy of the presented techniques in this paper. There is sufficient evidence to support the efficacy of nitinol staples used either alone or in conjunction with other constructs when it comes to fusion for foot and ankle indications. One of the concerns raised earlier on was that the use of nitinol staples results in an unacceptably high hardware failure rate. This was not the case in the most recent studies in the literature [13,21]. Furthermore, we underline that the orthopaedic literature on nitinol staples is only retrospective in nature, and therefore, caution should be exercised. Last but not least, we advise that caution should be exercised before making conclusions on the existing literature regarding nitinol staple fixation. This is because substantial heterogeneity exists in the studies addressing clinical and radiological outcomes.

### 3.9. Future Research Implications

First of all, we recommend that large-scale randomised trials be conducted to compare the impact of various staple combinations (i.e., different numbers and sizes of staples), especially for the Chopart joints that exhibit lower fusion rates [13,21]. Moreover, more data are needed to provide recommendations on the impact of patient factors, such as bone quality and graft augmentation, in addition to ideal staple characteristics, including arm/bridge length and number of implants per joint.

## 4. Conclusions

The use of nitinol staples has gained popularity in foot and ankle surgery recently, given the improvements in implant storage and simplicity of use. Overall, we claim that CCI internal fixation is a safe, reproducible, and reliable method when it comes to foot and ankle conditions, but it requires appropriate pre-operative planning, surgical training, and careful implantation. In our series, we demonstrated that primary fusion for Lisfranc injuries yields satisfactory short-term clinical and radiological results. We underline that more prospective papers are required to provide recommendations on ideal fixation characteristics, especially when it comes to hindfoot surgery.

## References

[1] Aiyer A., Russell N.A., Pelletier M.H., Myerson M., Walsh W.R. (2016). The Impact of Nitinol Staples on the Compressive Forces, Contact Area, and Mechanical Properties in Comparison to a Claw Plate and Crossed Screws for the First Tarsometatarsal Arthrodesis. Foot Ankle Spec..

[2] Schipper O.N., Ellington J.K. (2019). Nitinol Compression Staples in Foot and Ankle Surgery. Orthop. Clin. N. Am..

[3] Sands A., Zderic I., Swords M., Gehweiler D., Ciric D., Roth C., Nötzli C., Gueorguiev B. (2023). First Tarsometatarsal Joint Fusion in Foot—A Biomechanical Human Anatomical Specimen Analysis with Use of Low-Profile Nitinol Staples Acting as Continuous Compression Implants. Medicina.

[4] Russell N.A., Regazzola G., Aiyer A., Nomura T., Pelletier M.H., Myerson M., Walsh W.R. (2015). Evaluation of Nitinol Staples for the Lapidus Arthrodesis in a Reproducible Biomechanical Model. Front. Surg..

[5] Hoon Q.J., Pelletier M.H., Christou C., Johnson K.A., Walsh W.R. (2016). Biomechanical evaluation of shape-memory alloy staples for internal fixation-an in vitro study. J. Exp. Orthop..

[6] McKnight R.R., Lee S.K., Gaston R.G. (2019). Biomechanical Properties of Nitinol Staples: Effects of Troughing, Effective Leg Length, and 2-Staple Constructs. J. Hand Surg. Am..

[7] Curenton T.L., Davis B.L., Darnley J.E., Weiner S.D., Owusu-Danquah J.S. (2021). Assessing the biomechanical properties of nitinol staples in normal, osteopenic and osteoporotic bone models: A finite element analysis. Injury.

[8] Horner K., Summerhays B., Fiala K., Schweser K.M. (2023). Radiographic Evaluation of Isolated Continuous Compression Staples for Akin Osteotomy Fixation. J. Foot Ankle Surg..

[9] Carlsson Å.S., Onsten I., Besjakov J., Sturesson B. (1995). Isolated talo-navicular arthrodesis performed for non-inflammatory conditions blocks motion in healthy adjacent joints—A radiostereometric analysis of 3 cases. Foot.

[10] Carranza-Bencano A., Tejero S., Fernández Torres J.J., Del Castillo-Blanco G., Alegrete-Parra A. (2015). Isolated talonavicular joint arthrodesis through minimal incision surgery. Foot Ankle Surg..

[11] Zhao J.Z., Ingall E.M., Ritter Z., Kwon J.Y. (2022). Radiographically Occult Nonunions After Application of Nitinol Compression Staples: A Report of 3 Cases. Foot Ankle Int..

[12] Schipper O.N., Ford S.E., Moody P.W., Van Doren B., Ellington J.K. (2018). Radiographic Results of Nitinol Compression Staples for Hindfoot and Midfoot Arthrodeses. Foot Ankle Int..

[13] Dombrowsky A.R., Strickland C.D., Walsh D.F., Hietpas K., Conti M.S., Irwin T.A., Cohen B.E., Ellington J.K., Jones C.P., Shawen S.B. (2024). Nitinol Staple Use in Primary Arthrodesis of Lisfranc Fracture-Dislocations. Foot Ankle Int..

[14] Wang T., Pelletier M., Johnson J., Kline C., Walsh W., Lareau C., Safranski D. (2024). Biomechanical Performance of 4-Leg Sustained Dynamic Compression Staples in First Tarsometatarsal Arthrodesis. Foot Ankle Spec..

[15] Garlapaty A., Cook J.L., Bezold W., Schweser K. (2024). Activated nitinol compression staples are associated with favorable biomechanical properties for talonavicular arthrodesis. J. Orthop..

[16] Arumugam V., Ranjit S., Patel S., Welck M. (2023). What is the best fixation technique for isolated talonavicular arthrodesis?—A systematic review. Foot.

[17] Dock C.C., Freeman K.L., Coetzee J.C., McGaver R.S., Giveans M.R. (2020). Outcomes of Nitinol Compression Staples in Tarsometatarsal Fusion. Foot Ankle Orthop..

[18] O’Neil J.T., Abbasi P., Parks B.G., Miller S.D. (2020). Staple-Plate Plus Screw vs Screw Alone in Talonavicular Arthrodesis: A Cadaveric Biomechanical Study. Foot Ankle Int..

[19] Sleiman A., Bejcek C., Nestler A., Revelt N., Thuppal S., Mills A., Gardner M. (2023). The history of orthopaedic use of nitinol compression staples. Injury.

[20] Schafer K.A., Baldini T., Hamati M., Backus J.D., Hunt K.J., McCormick J.J. (2022). Two Orthogonal Nitinol Staples and Combined Nitinol Staple-Screw Constructs for a First Metatarsophalangeal Joint Arthrodesis: A Biomechanical Cadaver Study. Foot Ankle Int..

[21] Reddy A.R., Hampton H., Dzieza W.K., Toussaint R.J. (2024). Nitinol Compression Staples in Foot Orthopaedic Surgery: A Systematic Review. Foot Ankle Orthop..

